# Predictors of 30-day mortality in older adults with traffic-related injuries: Classical trauma scores, frailty, and laboratory markers in a retrospective study

**DOI:** 10.1097/MD.0000000000048423

**Published:** 2026-04-17

**Authors:** İskender Aksoy, Ali Altinbaş, Yiğit Şahin

**Affiliations:** a Department of Emergency Medicine, Faculty of Medicine, Giresun University, Giresun, Turkey; b Department of Anesthesiology, Faculty of Medicine, Giresun University, Giresun, Turkey.

**Keywords:** aged, frailty, mortality prediction, traffic accidents, trauma severity indices

## Abstract

Older adults are more vulnerable to trauma because of reduced physiological reserve, a higher burden of comorbidities, and increased frailty. However, traditional trauma scoring systems may not fully reflect these age-related factors. This study aimed to evaluate the predictive performance of classical trauma scoring systems and to examine the association of frailty and selected laboratory markers with 30-day mortality among older adults with traffic-related injuries. This retrospective study included patients aged ≥65 years who presented to a tertiary emergency department with traffic-related injuries between 2021 and 2024. Demographic, clinical, and laboratory data were recorded, along with frailty status assessed using the Clinical Frailty Scale (CFS). Trauma severity was evaluated using the Injury Severity Score (ISS), New Injury Severity Score (NISS), Trauma and Injury Severity Score (TRISS), Revised Trauma Score (RTS), Glasgow Age Pressure (GAP), Mechanism Glasgow Age Pressure (MGAP), and the Geriatric Trauma Outcome Score (GTOS). The primary outcome was 30-day mortality. Receiver operating characteristic (ROC) analysis and univariate logistic regression were performed to identify predictors of mortality. Among 325 patients (mean age: 73.4 years; 64.9% male), 30-day mortality was 4.9%. Chronic kidney disease was the only comorbidity significantly associated with mortality (OR: 7.69; *P* = .005). Mortality was associated with higher INR, lactate, and creatinine levels; lower platelet counts, Glasgow Coma Scale (GCS) scores, and systolic blood pressure; and increased respiratory rate. ISS, TRISS, and NISS demonstrated the highest discriminatory power (AUC > 0.99). Among laboratory parameters, lactate showed the strongest predictive performance (AUC = 0.910). In multivariate analysis, increased respiratory rate and higher NISS remained the only independent predictors of 30-day mortality. In this cohort of older adults with traffic-related trauma, increased respiratory rate and higher NISS were the main independent predictors of 30-day mortality. Frailty assessed by the Clinical Frailty Scale was not independently associated with short-term mortality. These findings suggest that, in high-energy traffic-related trauma, injury severity and acute physiological response may be more decisive for short-term outcomes than frailty status alone.

## 1. Introduction

Trauma is an important cause of morbidity and mortality in older adults. Older trauma patients have distinct clinical characteristics and often experience worse outcomes than younger adults because of age-related physiological vulnerability and comorbidity burden.^[1,2]^

Ageing is accompanied by multisystem physiological changes that reduce reserve capacity and may impair the response to acute stressors such as trauma. In addition, the high prevalence of comorbidities, polypharmacy, and anticoagulant use in older adults can make the clinical course of trauma more complex and less predictable. These factors may contribute to delayed recovery and worse outcomes even after injuries that appear less severe at initial assessment.^[1-3]^

Several trauma scoring systems have been developed to support clinical decision-making in the assessment of injury severity. The Injury Severity Score (ISS), Revised Trauma Score (RTS), New Injury Severity Score (NISS), and Trauma and Injury Severity Score (TRISS) were largely developed and validated in broader trauma populations, and their performance in older adults remains an area of ongoing discussion. Although TRISS incorporates age, conventional trauma scores may not fully account for geriatric-specific factors such as frailty, pre-injury functional status, and selected laboratory abnormalities.^[1,4,5]^

Frailty has become an increasingly important concept in geriatric trauma care, and current guidelines recommend its assessment as part of the overall evaluation of older trauma patients. Likewise, laboratory abnormalities such as elevated INR, thrombocytopenia, and lactate elevation may reflect physiological derangement and have been associated with adverse outcomes after trauma. However, the extent to which frailty and selected laboratory markers provide prognostic information beyond conventional trauma scores in acute high-energy trauma remains uncertain.^[1,3,6]^

In Turkey, published data on trauma in older adults have largely focused on general blunt trauma or mixed trauma populations, while studies specifically addressing traffic-related injuries in this age group remain limited. This gap is clinically relevant because trauma mechanisms may influence the prognostic importance of frailty, physiological variables, and laboratory findings. Accordingly, this retrospective study was designed to evaluate the predictive performance of conventional trauma scoring systems in older adults with traffic-related injuries and to examine the association of frailty and selected coagulation-related parameters with 30-day mortality.^[1,7,8]^

## 2. Materials and methods

### 2.1. Study design and population

This retrospective observational study was conducted in the emergency department of Giresun Training and Research Hospital, Giresun, Turkey. The study included patients aged 65 years and older who presented with traffic-related injuries between January 1, 2021 and December 31, 2024. After ethics committee approval was obtained in 2025, hospital records were retrospectively reviewed, and all eligible patients meeting the inclusion criteria were consecutively included. The study protocol was approved by the Scientific Research Ethics Committee of Giresun Training and Research Hospital (Approval No. BAEK-365; dated 25.06.2025) and was conducted in accordance with the principles of the Declaration of Helsinki.

### 2.2. Inclusion and exclusion criteria

Patients aged 65 years and older who presented to the emergency department due to traffic-related injuries during the study period were included. Patients younger than 65 years, those admitted for non-traffic-related trauma mechanisms (such as falls or penetrating injuries), and cases with incomplete clinical or laboratory data were excluded from the analysis.

### 2.3. Data collection

Following ethics committee approval obtained in 2025, hospital records of eligible patients presenting between January 2021 and December 2024 were retrospectively reviewed using a standardized data collection form. Demographic characteristics, vital signs at admission, clinical findings, laboratory parameters, trauma scores, frailty status, intensive care unit admission, length of hospital stay, and 30-day mortality outcomes were recorded. Thirty-day mortality data were determined using the national mortality database. ICU admission was considered a clinical outcome reflecting disease severity rather than a baseline predictor, and therefore was not interpreted as a causal determinant of mortality. All data were fully anonymized prior to analysis.

### 2.4. Definitions

Thirty-day mortality was defined as death from any cause occurring within 30 days after emergency department admission and was determined using the national mortality database. Intensive care unit (ICU) admission was defined as the need for ICU care at any point during the index hospitalization. Frailty status was assessed using the CFS, which classifies patients based on functional status and dependency levels prior to injury. Frailty was assessed retrospectively based on pre-injury functional status, comorbidity burden, and dependency levels documented in medical records at the time of admission.

### 2.5. Laboratory analysis

Laboratory parameters, including hemoglobin, lactate, creatinine, blood urea nitrogen, sodium, INR, and platelet count, were obtained from blood samples collected at the time of emergency department admission. Only initial laboratory values were used for analysis to reflect the patients’ physiological status at presentation.

### 2.6. Trauma scoring systems

Trauma severity was assessed using multiple validated scoring systems. The ISS and the NISS were used to quantify anatomical injury severity. The RTS is a physiological score based on Glasgow Coma Scale, systolic blood pressure, and respiratory rate. The TRISS combines anatomical and physiological parameters with age to estimate survival probability. The Glasgow Age Pressure (GAP) and Mechanism Glasgow Age Pressure (MGAP) scores incorporate age, Glasgow Coma Scale, systolic blood pressure, and injury mechanism for rapid mortality prediction. The Geriatric Trauma Outcome Score (GTOS) was used as a geriatric-specific model integrating age, injury severity, and transfusion requirements.

### 2.7. Statistical analysis

Statistical analyses were performed using IBM SPSS V25 (IBM Corp., Armonk). Data distribution was assessed using the Kolmogorov–Smirnov test. Comparisons between survivors and non-survivors were performed using the Independent Samples *t* test or chi-square test, as appropriate. Binary logistic regression analysis was initially conducted in a univariate manner to identify variables associated with 30-day mortality. Receiver operating characteristic (ROC) curve analysis was used to evaluate the discriminative performance of significant clinical, laboratory, and trauma scoring variables, with area under the curve (AUC) values and optimal cutoff points calculated using Youden index. Multivariate regression models incorporating multiple trauma scoring systems simultaneously were not constructed due to overlapping score components and the risk of multicollinearity. Continuous variables were expressed as mean (95% confidence interval) or median (minimum–maximum), as appropriate, while categorical variables were presented as frequencies and percentages. A 2-tailed *P* value of < .05 was considered statistically significant. For continuous variables, odds ratios represent the change in mortality risk associated with a 1-unit increase in the corresponding parameter.

## 3. Results

Among the 325 older adults with traffic-related injuries included in the study, 16 (4.9%) died within 30 days. Comparison of survivors and non-survivors showed no statistically significant differences in age, sex, or most comorbidities. However, the prevalence of chronic kidney disease was significantly higher in the mortality group (18.8% vs 2.9%, *P* = .001). Regarding laboratory parameters, non-survivors had significantly higher levels of creatinine (1.41 vs 1.01 mg/dL, *P* = .001), lactate (5.7 vs 1.2 mmol/L, *P* < .001), and INR (1.37 vs 1.05, *P* < .001), along with significantly lower platelet counts (182 vs 235 × 10^3^/µL, *P* = .003). In addition, systolic blood pressure (103 vs 130 mm Hg, *P* = .002), peripheral oxygen saturation (95.4% vs 97.8%, *P* < .001), and Glasgow Coma Scale scores (8 vs 15, *P* < .001) were markedly lower in the non-survivor group, whereas respiratory rate was significantly higher (15.1 vs 12.8 breaths/min, *P* < .001; Table 1).

**Table 1:** Comparison of baseline demographics and comorbidities between survivors and non-survivors.

	Total* (n = 325)	Survivors* (n = 309)	Mortality-in-30 d* (n = 16)	*P*
Age (yr)	73.4 (72.7–74.1)	73.4 (72.6–74.1)	73.3 (69.8–76.8)	.883
Female	114 (35.1)	109 (35.3)	5 (31.3)	.742
Male	211 (64.9)	200 (64.7)	11 (68.8)	
Hypertension	251 (77.2)	237 (76.7)	14 (87.5)	.315
Diabetes	81 (24.9)	76 (24.6)	5 (31.3)	.549
CAD	86 (26.5)	83 (26.9)	3 (18.8)	.473
CVD/demented	22 (6.8)	20 (6.5)	2 (12.5)	.349
Hypothyroidism	24 (7.4)	24 (7.8)	0 (0)	.247
Chronic kidney disease	12 (3.7)	9 (2.9)	3 (18.8)	**.001**
Asthma/COPD	68 (20.9)	66 (21.4)	2 (12.5)	.396
BUN	22 (21–23)	21.8 (20.8–22.8)	26.1 (19–33.1)	.256
Creatinine	1.03 (0.99–1.07)	1.01 (0.97–1.05)	1.41 (1.07–1.76)	**.001**
Urea	47.1 (44.9–49.2)	46.6 (44.4–48.8)	55.8 (40.6–70.9)	.259
Sodium	139.3 (139–139.6)	139.3 (139–139.7)	138.2 (136–140.4)	.224
Hemoglobin	12.8 (12.6–13)	12.9 (12.7–13.1)	11.6 (9.7–13.6)	.124
Platelet count	232 (225–240)	235 (227–242)	182 (146–219)	**.003**
Lactate	1.4 (1.2–1.6)	1.2 (1.1–1.3)	5.7 (2.8–8.5)	**<.001**
INR	1.06 (1.04–1.09)	1.05 (1.03–1.06)	1.37 (1–1.74)	**<.001**
Length of stay	3.9 (3–4.7)	3.4 (2.8–4)	13.4 (1–25.9)	**.024**
GCS†	15 (3–15)	15 (6–15)	8 (3–15)	**<.001**
Systolic BP (mm Hg)	129 (127–131)	130 (128–132)	103 (87–119)	**.002**
Respiratory rate	12.9 (12.7–13.1)	12.8 (12.6–12.9)	15.1 (13.4–16.7)	**<.001**
Pulse (/min)	76.7 (75.6–77.8)	76.2 (75.3–77.1)	86 (70.7–101.3)	.676
SpO_2_	97.7 (97.1–98.2)	97.8 (97.2–98.4)	95.4 (93.6–97.2)	**<.001**
ICU needs	32 (9.8)	21 (6.8)	11 (68.8)	**<.001**
CFS†	2 (1–7)	2 (1–7)	3 (1–5)	.145
ISS	6.3 (4.9–7.8)	3.8 (3.3–4.3)	55.9 (42–69.9)	**<.001**
NISS	6.5 (5–8)	3.9 (3.4–4.5)	56 (42.1–69.9)	**<.001**
RTS	7.7 (7.6–7.8)	7.8 (7.8–7.8)	5.3 (4.1–6.4)	**<.001**
TRISS	95.1 (93.3–96.9)	98.3 (98.1–98.6)	32.2 (10.8–53.6)	**<.001**
GAP	20.3 (20.1–20.6)	20.7 (20.6–20.8)	12.8 (9.5–16)	**<.001**
MGAP	19.8 (19.5–20)	20.1 (20–20.2)	13.3 (10.5–16)	**<.001**
GTOS	89.8 (86–93.7)	83.2 (81.6–84.9)	217.2 (182.5–251.8)	**<.001**

Bold values indicate statistical significance (*P* < .05).

BP = blood pressure, BUN = blood urea nitrogen, CAD = coronary artery disease, CFS = Clinical Frailty Scale, CI = confidence interval, COPD = chronic obstructive pulmonary disease, CVD = cerebrovascular disease, GAP = Glasgow Age Pressure, GCS = Glasgow Coma Scale, GTOS = Geriatric Trauma Output Score, ICU = intensive care unit, INR = international normalized ratio, ISS = Injury Severity Score, MGAP = Mechanism Glasgow Age Pressure, NISS = New Injury Severity Score, RTS = Revised Trauma Score, TRISS = Trauma and Injury Severity Score.

* Mean (95% CI)/n (%).

† Median (min–max).

ROC analysis demonstrated that classical trauma scoring systems such as ISS (AUC = 0.993), TRISS (AUC = 0.993), NISS (AUC = 0.992), GTOS (AUC = 0.984), and GCS (AUC = 0.962) provided excellent predictive accuracy for 30-day mortality. GAP (AUC = 0.912), MGAP (AUC = 0.906), and RTS (AUC = 0.873) also showed strong discriminative ability. In addition to these scoring models, laboratory and clinical variables including lactate (AUC = 0.910), INR (AUC = 0.772), creatinine (AUC = 0.736), respiratory rate (AUC = 0.740), and low SpO_2_ (AUC = 0.759) contributed significantly to mortality prediction. Notably, GTOS scores were significantly higher in non-survivors than in survivors (217.2 vs 83.2; Table 2).

**Table 2:** Diagnostic performance of laboratory parameters in predicting 30-day mortality (ROC analysis).

	AUC (95% CI)	*P*	Cutoff	Sensitivity	Specificity	PPV	NPV	Accuracy
Creatinine (↑)	0.736 (0.607–0.865)	**.001**	1.115	62.5 (35.4–84.8)	72.8 (67.5–77.7)	10.6 (7.3–15.4)	97.4 (95.2–98.6)	72.3 (67.1–77.1)
Platelet count (↓)	0.720 (0.578–0.862)	**.003**	205.5	68.8 (41.3–89.0)	65.7 (60.1–71.0)	9.4 (6.7–13.0)	97.6 (95.1–98.8)	65.9 (60.4–71.0)
Lactate (↑)	0.910 (0.840–0.979)	**<.001**	1.525	81.3 (54.4–95.9)	81.6 (76.8–85.7)	18.8 (14.1–24.1)	98.8 (96.8–99.6)	81.5 (76.9–85.6)
INR (↑)	0.772 (0.649–0.895)	**<.001**	1.085	68.8 (41.3–89.0)	70.9 (65.5–75.9)	10.9 (7.8–15.1)	97.8 (95.5–98.9)	70.8 (65.5–75.7)
GCS (↓)	0.962 (0.892–1.000)	**<.001**	14.5	93.8 (69.8–99.8)	96.1 (93.3–98.0)	55.6 (41.4–68.8)	99.7 (97.8–99.9)	96.0 (93.3–97.9)
Systolic BP, mm Hg (↓)	0.728 (0.564–0.892)	**.002**	122.5	68.8 (41.3–89.0)	55.7 (49.9–61.3)	7.4 (5.3–10.3)	97.2 (94.3–98.6)	56.3 (50.7–61.8)
Respiratory rate (↑)	0.740 (0.573–0.907)	**.001**	13.5	68.8 (41.3–89.0)	68.9 (63.5–74.1)	10.3 (7.3–14.2)	97.7 (95.4–98.9)	68.9 (63.6–73.9)
SpO_2_ (↓)	0.759 (0.622–0.896)	**<.001**	97.5	56.3 (29.9–80.3)	78.6 (73.6–83.1)	12.0 (7.8–18.1)	97.2 (95.2–98.4)	77.5 (72.6–82.0)
ISS (↑)	0.993 (0.982–1.000)	**<.001**	15	93.8 (69.8–99.8)	97.1 (94.5–98.7)	62.5 (46.4–76.3)	99.7 (97.8–99.9)	96.9 (94.4–98.5)
NISS (↑)	0.992 (0.981–1.000)	**<.001**	15	93.8 (69.8–99.8)	96.8 (94.1–98.4)	60.0 (44.6–73.7)	99.7 (97.8–99.9)	96.6 (94.0–98.3)
RTS (↓)	0.873 (0.745–1.000)	**<.001**	7.3724	75.0 (47.6–92.7)	99.4 (97.7–99.9)	85.7 (59.4–96.1)	98.7 (97.1–99.5)	98.2 (96.0–99.3)
TRISS (↓)	0.993 (0.983–1.000)	**<.001**	95.55	93.8 (69.8–99.8)	97.4 (94.5–98.7)	62.5 (46.4–76.3)	99.7 (97.8–99.9)	96.9 (94.4–98.5)
GAP (↓)	0.912 (0.811–1.000)	**<.001**	20.5	87.5 (61.7–98.5)	86.4 (82.1–90.0)	25.0 (19.2–31.8)	99.3 (97.3–99.8)	86.5 (82.3–90.0)
MGAP (↓)	0.906 (0.779–1.000)	**<.001**	19.5	87.5 (61.7–98.5)	96.8 (94.1–98.4)	58.3 (42.5–72.6)	99.3 (97.6–99.8)	96.3 (93.6–98.1)
GTOS (↑)	0.984 (0.963–1.000)	**<.001**	112.25	93.8 (69.8–99.8)	94.2 (91.0–96.5)	45.5 (34.3–57.0)	99.7 (97.8–99.9)	94.2 (91.0–96.4)

Bold values indicate statistical significance (*P* < .05).

AUC = area under the curve, BP = blood pressure, GAP = Glasgow Age Pressure, GCS = Glasgow Coma Scale, GTOS = Geriatric Trauma Output Score, INR = international normalized ratio, ISS = Injury Severity Score, MGAP = Mechanism Glasgow Age Pressure, NISS = New Injury Severity Score, NPV = negative predictive value, PPV = positive predictive value, ROC = receiver operating characteristic, RTS = Revised Trauma Score, TRISS = Trauma and Injury Severity Score.

Univariate logistic regression analysis identified several factors significantly associated with 30-day mortality, including elevated INR (OR: 41.09, *P* = .002), intensive care unit admission (OR: 30.17, *P* < .001), low RTS score (OR: 36.95, *P* < .001), elevated creatinine (OR: 3.72, *P* = .002), elevated lactate (OR: 2.52, *P* < .001), low platelet count (OR: 1.01, *P* = .003), low GCS (OR: 2.57, *P* < .001), low systolic blood pressure (OR: 1.08, *P* < .001), increased respiratory rate (OR: 2.00, *P* < .001), high ISS (OR: 1.36, *P* < .001), high GTOS (OR: 1.09, *P* < .001), and low GAP, MGAP, and TRISS scores (Table 3).

**Table 3:** Univariate logistic regression analysis for predictors of 30-day mortality.

	OR	95% CI	*P*
Chronic kidney disease	7.692	1.860–31.815	**.005**
Creatinine (↑)	3.716	1.615–8.551	**.002**
Platelet count (↓)	1.013	1.004–1.021	**.003**
Lactate (↑)	2.524	1.664–3.828	**<.001**
INR (↑)	41.091	3.784–446.274	**.002**
ICU needs	30.171	9.590–94.926	**<.001**
GCS (↓)	2.574	1.705–3.886	**<.001**
Systolic BP, mm Hg (↓)	1.077	1.046–1.108	**<.001**
Respiratory rate (↑)	1.997	1.495–2.669	**<.001**
ISS (↑)	1.357	1.162–1.585	**<.001**
NISS (↑)	1.317	1.154–1.503	**<.001**
RTS (↓)	36.950	5.316–256.829	**<.001**
TRISS (↓)	1.230	1.090–1.388	**.001**
GAP (↓)	2.166	1.540–3.048	**<.001**
MGAP (↓)	2.270	1.577–3.267	**<.001**
GTOS (↑)	1.093	1.045–1.144	**<.001**

Bold values indicate statistical significance (*P* < .05).

BP = blood pressure, CI = confidence interval, GAP = Glasgow Age Pressure, GCS = Glasgow Coma Scale, GTOS = Geriatric Trauma Output Score, ICU = intensive care unit, INR = international normalized ratio, ISS = Injury Severity Score, MGAP = Mechanism Glasgow Age Pressure, NISS = New Injury Severity Score, OR = odds ratio, RTS = Revised Trauma Score, TRISS = Trauma and Injury Severity Score.

Variables found to be significant in univariate analysis were subsequently entered into a multivariate logistic regression model using a backward stepwise approach. In the final model, increased respiratory rate (adjusted OR: 2.54, *P* = .017) and higher NISS (adjusted OR: 1.46, *P* = .002) values remained independently associated with 30-day mortality (Table 4). These findings indicate that increased respiratory rate and higher NISS were the main independent predictors of 30-day mortality in this cohort, while the other significant variables identified in univariate analyses did not remain independently associated with mortality in the final model.

**Table 4:** Multivariate logistic regression analysis identifying independent predictors of 30-day mortality using a backward stepwise approach.

	OR	95% CI	*P*
Respiratory rate (↑)	2.537	1.184–5.436	**.017**
NISS (↑)	1.455	1.147–1.847	**.002**

Bold values indicate statistical significance (*P* < .05).

CI = confidence interval, NISS = New Injury Severity Score, OR = odds ratio.

## 4. Discussion

Mortality rates among older trauma patients are substantially higher compared with younger populations, largely attributable to increased physiological frailty, a greater burden of comorbidities, and diminished compensatory reserve with advancing age. Previous reports indicate that 30-day mortality in patients over 65 years may reach up to 14.9%,^[2]^ whereas in our cohort the rate was 4.9%. Similarly, data from North America demonstrate that in this age group, 1-year mortality may rise to 26.5%, and 3-year mortality to 36.3%.^[9]^ The lower mortality observed in our study may be explained by the relatively less severe injuries sustained by our patient population and the implementation of effective early intervention strategies. Additionally, the exclusive inclusion of traffic accident cases may have contributed to the lower mortality rate, as some studies have suggested that high-energy traumas may be associated with better long-term survival compared with low-energy injuries.^[10]^ To our knowledge, this study is among the few to comprehensively evaluate frailty, coagulation parameters, and multiple trauma scoring systems together in older adults presenting with traffic-related injuries, while also showing that the main independent predictors of 30-day mortality were injury severity and respiratory rate.

In addition, the quality of hospital-based care and the adoption of multidisciplinary approaches are crucial determinants of mortality. The literature indicates that treatment of older trauma patients in specialized trauma centers significantly reduces mortality rates.^[11]^ Early identification of ICU needs and timely patient transfers are also recognized as critical factors directly influencing survival.^[12]^ The findings of our study further underscore the importance of rapid assessment and appropriate management in optimizing outcomes for older trauma patients.

In our study, only chronic kidney disease (CKD) was found to be significantly associated with 30-day mortality. The presence of CKD increased the risk of death nearly eightfold and emerged as an independent predictor. This finding highlights the critical role of diminished renal reserve in determining clinical outcomes under conditions of hypoperfusion, inflammation, and metabolic stress following trauma. Consistent with our results, the existing literature has also reported that the presence of chronic kidney disease negatively impacts survival in trauma patients^[6,13]^

The prognostic significance of renal dysfunction is not limited to chronic disease. Acute kidney injury, particularly when secondary to trauma, has been reported as the third most common cause of death.^[14]^ Therefore, early evaluation of renal function and the avoidance of nephrotoxic agents in high-risk patients represent important clinical strategies for reducing mortality.

In our study, lactate level emerged as the biomarker with the highest discriminative power for predicting mortality (AUC = 0.910). This finding indicates that tissue hypoperfusion and the shift to anaerobic metabolism exert systemic effects at an early stage. The literature has similarly demonstrated that both lactate concentration and clearance are strongly associated with survival.^[15,16]^ In older patients, the stronger association between elevated lactate levels and increased mortality may be explained by their limited physiological reserve.

Among coagulation parameters, elevated INR and thrombocytopenia were also found to be significantly associated with mortality. ROC analyses identified INR > 1.085 and platelet count < 205.5 × 10^3^/µL as meaningful cutoff values with strong discriminative ability. These findings suggest that trauma-induced coagulopathy exerts a substantial impact on prognosis in older patients. Particularly in those with traumatic brain injury, elevated INR and reduced platelet count have been recognized as powerful predictors of mortality.^[5]^ Consistently, Hollands et al also reported that coagulopathy and thrombocytopenia were closely linked to early mortality in older trauma patients.^[6]^ In our study, elevated creatinine was additionally found to be significant, reflecting not only the presence of comorbid CKD but also the risk of acute renal dysfunction.

In our study, the classical trauma scoring systems ISS, NISS, and TRISS demonstrated strong predictive ability for 30-day mortality. TRISS, in particular, emerged as one of the most powerful models, with an accuracy exceeding 97%. While ROC analysis demonstrated strong discriminatory performance for several trauma scores and laboratory parameters, multivariate regression analysis was required to identify variables independently associated with mortality. Nevertheless, the GTOS, developed specifically for older populations, also showed significant discriminative capacity and, owing to its ease of calculation in clinical practice, appears to be an important complementary tool. Previous studies have reported that GTOS performs comparably to TRISS, and in certain subgroups, it may even exhibit higher sensitivity.^[17-19]^ In multivariate analysis, respiratory rate and NISS emerged as independent predictors of 30-day mortality, suggesting that the combination of acute physiological response and anatomical injury burden plays a key role in outcomes among older trauma patients. This finding suggests that both anatomical injury burden and acute physiological response should be considered in the early assessment of this high-risk population. These findings are consistent with the recent study by Kaya et al (2025), who compared multiple trauma scoring systems in emergency department patients with traffic-related multiple trauma and reported that TRISS and GCS exhibited the highest discriminative ability for mortality prediction, while ISS and NISS also demonstrated robust performance. The concordance between their results and our findings underscores the continued reliability of classical trauma scores in traffic-related injuries. However, differences in patient age and population characteristics between the 2 studies highlight the potential impact of geriatric physiology on trauma score performance. In older patients, diminished physiological reserve, comorbidity burden, and altered responses to injury may further modify the prognostic value of these scoring systems.^[20]^

The applicability of classical scoring systems in the older adult population has long been a matter of debate. Although TRISS incorporates age as a variable, it does not account for geriatric-specific parameters such as frailty, coagulopathy, or functional independence.^[4]^ Indeed, Mörs et al reported that older trauma patients exhibit a more pronounced proinflammatory response and higher mortality rates, suggesting that conventional scores alone may be insufficient in this population.^[21]^

The MGAP and GAP scores, based on age, GCS, and vital signs, offer the advantage of rapid applicability. Previous studies have shown that these scores may demonstrate predictive performance comparable to, or even better than, the RTS.^[22,23]^ Our findings support this evidence, indicating that scores such as GAP and MGAP contribute to rapid decision-making, particularly during the triage phase.

Our study clearly demonstrated a strong association between vital parameters: including GCS, systolic blood pressure, respiratory rate, and SpO_2_ – and mortality. In particular, the GCS exhibited an almost perfect discriminative ability with an AUC of 0.962, underscoring the critical role of neurological status in prognostication. The literature similarly reports that low GCS values in older trauma patients may lead to diagnostic undertriage, highlighting the need for careful clinical evaluation.^[24,25]^

A systolic blood pressure of <110 mm Hg has been significantly associated with increased mortality in older patients.^[26]^ An elevated respiratory rate and reduced SpO_2_ values particularly reflect traumatic hypoxia and shock, making them important predictors of early mortality.^[27,28]^ Our findings support the notion that these parameters serve as indispensable clinical tools for rapid and effective triage.

In our study, the CFS score was not significantly associated with mortality. This finding may be explained by the relatively homogeneous sample, the limited number of patients in the mortality group, and the mechanism-specific nature of the cohort, which included only traffic-related trauma. The literature suggests that frailty serves as a more prominent prognostic marker in low-energy trauma, whereas in high-energy trauma, the severity of injury and physiological derangement are more decisive factors.^[1,3]^

Several cohort studies have shown that frailty does not necessarily reflect short-term mortality but is more closely associated with complication rates, long-term functional decline, and quality of life.^[28,29]^ Therefore, scales such as the CFS may be more effective in rehabilitation and long-term care planning rather than in the acute management of trauma.^[29,30]^

### 4.1. Strengths and limitations

This study represents one of the few investigations in Turkey focusing specifically on traffic accident patients aged over 65 years. Most studies in the literature address older trauma populations in general, encompassing falls, penetrating injuries, and other mechanisms. By restricting our analysis to traffic accidents, this study provides epidemiological and prognostic data unique to this high-risk subgroup.

Another strength of the study lies in its simultaneous evaluation of multiple trauma scoring systems. The performances of ISS, NISS, TRISS, GAP, MGAP, and GTOS were directly compared, while frailty and coagulation parameters were additionally analyzed. This comprehensive approach highlighted the predictive strengths of classical trauma scores in older adults and allowed the simultaneous evaluation of geriatric-specific and laboratory-related variables such as GTOS, CFS, INR, and platelet count.

Our study also underscored the clinical contribution of laboratory parameters. The identification of lactate, INR, platelet count, and creatinine levels as significant predictors of mortality indicates that readily available laboratory tests in the emergency setting may be effectively used for risk stratification. This finding is consistent with previous reports emphasizing their prognostic utility, particularly in older and frail patients.

Finally, the study provides practical insights with direct implications for emergency department practice. Early assessment of vital parameters such as GCS, systolic blood pressure, respiratory rate, and SpO_2_, together with trauma severity scores and selected laboratory findings, may assist clinicians in the initial risk assessment of older adults with traffic-related trauma. These data may help support clinical judgment and resource planning in the emergency setting.

Nonetheless, several limitations should be acknowledged. First, the single-center and retrospective design limits the generalizability of the findings. Retrospective studies in older trauma populations are particularly prone to incomplete records and heterogeneous data structures, as noted in the literature.

Second, the study exclusively included traffic accident-related injuries. As such, it does not fully represent the broader older trauma population, in which falls predominate. However, given that traffic accidents constitute a subgroup with particularly high mortality risk, this focus also represents a unique aspect of the study. The results, therefore, should not be generalized to all trauma mechanisms.

Third, the trauma scores and laboratory parameters were based on values obtained at the time of admission. Hemodynamic changes and laboratory fluctuations occurring during the clinical course could not be captured. Prior research suggests that dynamic monitoring parameters may also influence prognosis in older trauma patients. Furthermore, the relatively low number of mortality events (n = 16) may have limited the robustness of regression analyses and may increase the risk of overfitting, particularly in models demonstrating very high discriminative performance.

Lastly, mortality outcomes were limited to 30 days. Long-term survival was not assessed, which constitutes an additional limitation of this study.

## 5. Conclusion

This study evaluated the performance of classical trauma scoring systems in predicting 30-day mortality among older adults with traffic-related injuries. TRISS, ISS, and NISS showed high predictive performance, and GTOS also demonstrated clinical relevance. In the multivariate model, increased respiratory rate and higher NISS were the main independent predictors of 30-day mortality, whereas the other clinical and laboratory variables did not retain independent significance.

These findings suggest that, in older adults with high-energy traffic-related trauma, injury severity and acute physiological response may be more decisive for short-term mortality than frailty status alone. Frailty assessed by the Clinical Frailty Scale was not independently associated with 30-day mortality in this cohort. Our study adds to the limited literature on traffic-related geriatric trauma and highlights the need for larger, multicenter, prospective studies to further clarify the role of frailty and laboratory markers in different trauma mechanisms.

## Author contributions

**Conceptualization:** İskender Aksoy.

**Data curation:** İskender Aksoy, Yiğit Şahin.

**Formal analysis:** İskender Aksoy.

**Investigation:** İskender Aksoy, Ali Altinbaş, Yiğit Şahin.

**Methodology:** İskender Aksoy, Ali Altinbaş, Yiğit Şahin.

**Supervision:** Ali Altinbaş.

**Validation:** İskender Aksoy, Ali Altinbaş, Yiğit Şahin.

**Visualization:** İskender Aksoy.

**Writing – original draft:** İskender Aksoy.

**Writing – review & editing:** Ali Altinbaş, Yiğit Şahin.
